# Obesity under the moonlight of c-MYC

**DOI:** 10.3389/fcell.2023.1293218

**Published:** 2023-12-05

**Authors:** Yulia A. Nevzorova, Francisco Javier Cubero

**Affiliations:** ^1^ Department of Immunology, Ophthalmology and ENT, Complutense University School of Medicine, Madrid, Spain; ^2^ Centro de Investigación Biomédica en Red de Enfermedades Hepáticas y Digestivas (CIBEREHD), Madrid, Spain; ^3^ Instituto de Investigación Sanitaria Gregorio Marañón (IiSGM), Madrid, Spain

**Keywords:** c-Myc, obesity, MASLD, gut-liver axis, T2DM

## Abstract

The moonlighting protein c-Myc is a master regulator of multiple biological processes including cell proliferation, differentiation, angiogenesis, apoptosis and metabolism. It is constitutively and aberrantly expressed in more than 70% of human cancers. Overwhelming evidence suggests that c-Myc dysregulation is involved in several inflammatory, autoimmune, metabolic and other non-cancerous diseases. In this review, we addressed the role of c-Myc in obesity. Obesity is a systemic disease, accompanied by multi-organ dysfunction apart from white adipose tissue (WAT), such as the liver, the pancreas, and the intestine. c-Myc plays a big diversity of functions regulating cellular proliferation, the maturation of progenitor cells, fatty acids (FAs) metabolism, and extracellular matrix (ECM) remodeling. Moreover, c-Myc drives the expression of a wide range of metabolic genes, modulates the inflammatory response, induces insulin resistance (IR), and contributes to the regulation of intestinal dysbiosis. Altogether, c-Myc is an interesting diagnostic tool and/or therapeutic target in order to mitigate obesity and its consequences.

## Introduction

Obesity is defined by the World health organization (WHO) as an excessive fat accumulation that impairs health with a diagnosis of a body mass index (BMI) ≥30 kg/m^2^.

Since 1975, the global prevalence of obesity has almost tripled and has continued to increase at an epidemic rate. In the past decades, obesity has been revisited and now it is considered a multisystemic disease affecting many multiple organs. Since it is a chronic, systemic and relapsing disorder, obesity triggers a significant number of metabolic disorders and co-morbidities. Obesity considerably elevates the risk of suffering type 2 diabetes mellitus (T2DM), metabolic-associated steatotic liver disease (MASLD), hypertension, myocardial infarction, stroke, obstructive sleep apnoea, dementia, osteoarthritis, and several cancers, thereby decreasing both quality and life expectancy (Bluher, 2019; Gjermeni et al., 2021; Lin and Li, 2021).

Recent studies revealed a clear link between obesity and urbanisation, demonstrating the crucial role that environment plays in the development of this disease. However, the considerable variation in body weight between individuals, further suggests that obesity is influenced by complex interactions between environmental developmental, behavioural, epigenetics and genetic stimuli (Thaker, 2017; Zaiou, 2022).

In recent years, it has become evident that the highly pleiotropic, multifunctional super-transcription factor (TF) c-Myc controls a variety of cellular functions by targeting up to 15% of all genes, with broad effects on cell proliferation, differentiation, apoptosis, angiogenesis, adhesion and metabolism (Dang, 2012). Different cytokines and hormones can promote stabilization of c-Myc protein levels and subsequently activate nuclear transactivation of c-Myc-dependent target genes. Among these, genes involved in cell cycle regulation such as cyclins D1, D2, B1, cyclin-dependent kinase 4 (CDK4) and p21, p27 inhibitors of CDK (Dang, 2012).

Additionally, c-Myc also attenuates the differentiation of a great number of cell types during development, thus preserving the “stemness” of these cells (Leon et al., 2009). In spite of its association with cell proliferation and differentiation, c-Myc also promotes apoptosis and provides an additional level of regulation against uncontrolled cell growth or when the growth factors are limited (McMahon, 2014; Madden et al., 2021).

Metabolism is regulated by c-Myc through enolase A, lactate dehydrogenase A, phosphofructokinase, hexokinase II, and glucose transporter I. c-Myc expression stimulates glutaminolysis and glycolysis (Goetzman and Prochownik, 2018). Both pathways promote cellular proliferation by increasing the synthesis of nucleotides, ATP and fatty acids (FA) that serve as building blocks for cells (Dang, 2013).

Through the activation of peroxisome proliferator-activated receptor gamma coactivator 1 (PGC-1), protein kinases, mitochondrial TF, and mitochondrial receptors, c-Myc encourages mitochondrial biogenesis and enhances mitochondrial function (Dang, 2013).

In order to increase cell mass before division, c-Myc stimulates global protein expression, via the activation of RNA polymerase I, II, and III and of genes that participate in ribosomal, tRNA and rRNA biosynthesis (Dang, 2013; Rosselot et al., 2021).

Therefore, c-Myc carries out a great number of biological functions that are essential for survival, expansion, and normal cell function. Generally, c-Myc expression is tightly regulated; however, its deregulation is often observed in human cancer and is considered a poor prognostic factor. Therefore, it was termed “the oncogene from hell”, given its ability to induce genomic instability, accelerate tumour progression and coordinate the crosstalk with microenvironment, thus inducing tumor growth (Whitfield and Soucek, 2012).

Additionally to its role in carcinogenesis, c-Myc appears to be involved in the control of multiple metabolic pathways from glycolysis and glutaminolysis, to nucleotide and lipid synthesis across many different cell types, especially as almost all cells basally express metabolic genes (Stine et al., 2015). Emerging evidence also suggests that c-Myc is pivotal in driving the expression of a broad range of immune cell metabolism, regulating their development, differentiation, activation and coordination of metabolic programs to support immune functions (Gnanaprakasam and Wang, 2017). As a master regulator of immunity and metabolism, c-Myc is implicated in autoimmune, inflammatory, metabolic and other non-cancerous disorders (Zheng et al., 2017), even though this is still a poorly understood topic with a huge unmet need for preclinical and clinical research (Madden et al., 2021).

In this review, we aimed to highlight and summarize the potential roles of multifunctional moonlighting c-Myc in obesity and its related metabolic diseases, including T2DM and MASLD. Indeed, the complexity of the etiopathogenesis of obesity is responsible for the dysfunction of multiple tissues and organs, including the white adipose tissue (WAT), pancreas, liver and intestine. All of the above makes the understanding of the complex role of c-Myc in the development of obesity increasingly challenging. Here, we provide a comprehensive view of c-Myc-related disturbances present in obesity and their direct and indirect effects on the different organs of the body.

### WAT –holding the key of obesity

White adipose tissue (WAT) is crucial for the regulation of lipid homeostasis and energy balance. In a healthy state, WAT serves a variety of purposes, including storing energy as fat, protecting vital organs, and assisting with the endocrine system and immune response. The adipose tissue consists of adipocytes, endothelial cells, fibroblasts, immune cells, and adipose stem cells (ASCs) (Richard et al., 2000).

Obesity is the result of storing excess energy intake, thus bringing about an enlargement of the adipose tissue. Diet, genetics, and their interaction contribute to obesity (Jo et al., 2009). The expansion of the WAT associated with obesity is linked to an elevation of the adipogenesis activity. The coordinated activation of TFs and epigenetic modifications control the lipogenic and adipogenic programmes. The complicated regulatory mechanisms, however, are not yet fully understood (Longo et al., 2019).

The increase in size of existing adipocytes (hypertrophy) or in number (hyperplasia) (Jo et al., 2009) is characteristic of WAT expansion. An imbalance in caloric intake *versus* expenditure leads to the accumulation of hypertrophic and dysfunctional adipocytes. While hypertrophic growth is more closely linked to obesity-associated metabolic complications, expansion through hyperplasia is associated with a benign metabolic profile. Numerous adipogenic processes, such as the proliferation, recruitment, and/or differentiation of new fat cells, are responsible for mediating hyperplasic WAT, whereas hypertrophy is mainly governed by size increase of already present adipocytes (Choe et al., 2016). Adipogenesis and transition of adipose tissue mesenchymal stem cells to mature adipocytes is regulated by an extensive cooperative network of transcription factors (TFs), that control the expression of dozens of downstream protein-coding genes and long noncoding RNAs (Ambele and Pepper, 2017; Bjork et al., 2021).

The stromal vascular fraction of subcutaneous WAT is the source of human ASCs. ASCs are multipotent, fibroblast-like mesoderm lineage cells with the ability to differentiate into multiple lineages, much like bone marrow-derived mesenchymal stem cells. In adult WAT, the turnover of adipocytes at approximately a rate of ∼10% of cells per year maintains the balance between cell renewal and death. In accordance to several studies, ASCs play an essential role in the development of obesity and obesity-related metabolic disorders (Hajer et al., 2008). Furthermore, ASC quantity and function can also change in an obese state due to adipocyte dysfunction, which can result in abnormal adipose tissue remodeling and affect the microenvironment of expanded WAT (Choe et al., 2016).

The positive energy balance provokes the proliferation of ASCs, and when adipocytes reach a volume limit, the newly formed ASCs are utilized for *de novo* adipogenesis to further increase energy storage capacity of adipose tissue (Wang et al., 2013; Jeffery et al., 2015). The capacity of mature white adipocytes to dedifferentiate into multipotent ASCs is another feature. The functions of ASCs change in an obese condition, which causes a rise in the production of white fat and a whitening of thermogenic brown and beige fat (Shin et al., 2020). Additional research is still required to uncover the mechanism how ASCs generate new adipocytes in obesity, and the impact of environmental and genetic factors on this response.

It has been shown that c-Myc is positive regulator of ASCs fate and plays a crucial role in regulating adipocyte differentiation. Deregulated c-Myc expression prevents adipocytes and other cell types from achieving terminal differentiation (Spalding et al., 2008).

The expression of the c-Myc protein and transcript rises during the early stages of ASC differentiation and is thought to be involved in adipogenesis and the maintenance of a terminal phenotype. siRNA mediated knockdown of c-Myc in ASCs lead to inhibition of adipogenesis and dysregulation of pathways related to cytoskeletal remodelling and cell adhesion. These findings show that c-Myc is essential for driving multipotent ASCs into the adipogenic lineage (Deisenroth et al., 2014).

Overexpression of c-Myc in 3T3-L1 preadipocytes facilitates normal expression of early response regulators CCAAT/enhancer binding proteins C/EBPβ and C/EBPδ during the course of differentiation. However, the expression of downstream regulators, C/EBPα, peroxisome proliferator-activated receptor γ2 (PPARγ2), and later markers of differentiation is suppressed (Heath et al., 2000b). This suggests that c-Myc may act by blocking C/EBPβ- and C/EBPδ-directed activation of C/EBPα and PPARγ2 expression and demonstrates that c-Myc specifically inhibits the terminal stages of the adipogenic program. However, the particular molecular mechanism is not fully understood, yet (Heath et al., 2000b; Deisenroth et al., 2014). Interestingly, comparable outcomes were shown in hematopoietic stem cells, where c-Myc maintains the balance between stem cell differentiation and self-renewal via the regulation of cell-ECM interactions (Wilson et al., 2004). Importantly, c-Myc’s role in cell cycle progression and transformation is functionally different from the way it induces the suppression of adipocyte differentiation (Heath et al., 2000a).

There is still a lack of clarity in the associated signaling pathways that could be used as potential therapeutic targets for c-Myc-driven adiposity. The mammalian Sirtuins (SIRT1–7) are a family of conserved NAD^+^-dependent protein deacetylases. A growing body of evidences has shown that Sirtuins and their prominent substrates participate in a variety of physiological and pathological processes, including cell cycle regulation, glucose and lipid metabolism, mitochondrial biogenesis and function, energy homeostasis insulin action and inflammatory responses (Guarente, 2006; Chen et al., 2022). The nuclear sirtuins (SIRT1, SIRT6, and SIRT7), the mitochondrial sirtuins (SIRT3, SIRT4, and SIRT5), and the cytosolic sirtuin (SIRT2) regulate diverse metabolic functions. For example, SIRT1 controls several physiological processes in adipose tissue, such as inflammatory responses, mitochondrial biogenesis, cellular senescence, and apoptosis/autophagy (Hwang et al., 2013). SIRT2 regulates adipocyte development, gluconeogenesis, insulin action, and inflammatory responses (Gomes et al., 2015). By regulating mitochondrial biogenesis and function, SIRT3 plays regulating roles in a variety of metabolic processes, including acetate metabolism and thermogenesis (Shi et al., 2010).

It has been reported that Sirtuins are affected by HFD and environmental stress (Jokinen et al., 2017). In WAT of mice, pigs, and humans, restriction of nutrients causes SIRT1 upregulation, leads to changes in NAD^+^ levels and act by deacetylating forkhead box protein (FOXO), peroxisome proliferator activated receptor gamma coactivator1 (PGC-1α), PPARγ and Nuclear factor kappa b (NF-κB). In contrast obesity is linked to lower levels of SIRT1(Lakhan and Kirchgessner, 2011). For instance, in comparison to obese women, thin women exhibited over two times the SIRT1 expression (Pedersen et al., 2008). In WAT of obese HFD-fed mice and db/db mice SIRT1 expression is low (Chalkiadaki and Guarente, 2012). Mechanistically, adipogenesis is boosted when SIRT1 is downregulated in WAT. In contrast, adipogenesis is suppressed and lipolysis is promoted when SIRT1 expression in WAT is high (Picard et al., 2004).

Whole-body SIRT1 overexpression protects against genetically-induced obesity and from age-induced glucose intolerance (Herranz et al., 2010). Genetic deletion of SIRT1 from adipocytes leads to increases adiposity, exaggerated insulin resistance, glucose intolerance, inflammation and predisposes to metabolic disfunction in mice on short-term HFD (Mayoral et al., 2015). Less inflammation, improved glucose tolerance, and virtually total protection against hepatic steatosis are the advantages of SIRT1 over-expression, indicating that SIRT1 is crucial in preventing the adverse metabolic effects of obesity (Banks et al., 2008). Furthermore, SIRT1 activation causes weight loss without a reduction in calorie intake (Feige et al., 2008; Pfluger et al., 2008).

3T3-L1 preadipocytes from SIRT1-deficient mice differentiate into tiny, dysfunctional, inflamed, hyperplastic adipocytes with increased proliferative potential. Remarkably, in SIRT1-silenced preadipocytes c-Myc is hyperacetylated and activated leading to, uncontrolled cell proliferation and the development of hyperplastic, defective adipocytes. Additionally, SIRT1-silenced human SW872 preadipocytes and proliferating SIRT1 knockdown MEFs have shown the increased proliferation. Preadipocytes’ inability to undergo hyperplasia when both SIRT1 and c-Myc expression were simultaneously reduced suggests that SIRT1 controls adipocyte hyperplasia through c-Myc regulation. Therefore, the SIRT1/c-Myc axis controls the quantity of adipocytes and their functional integrity (Abdesselem et al., 2016). It seems that c-Myc and SIRT1 form a negative-feedback loop that inhibits c-Myc-induced cellular transformation. On one hand, c-Myc binds to the SIRT1 promoter and induces SIRT1 expression. However, SIRT1 in turn deacetylates and downregulates c-Myc, resulting in decreased c-Myc stability, reduced target gene expression and cellular transformation (Yuan et al., 2009). The functional relationships between SIRT1 and c-Myc in the control of adipocyte proliferation and differentiation will be intriguing to further explore.

Another surprising and important functional link has been described between c-Myc and mammalian target of rapamycin (mTOR) (Pourdehnad et al., 2013). mTOR regulates eukaryotic cell growth and metabolism in response to environmental variables including nutrition and growth factors. It is an important regulator of lipid metabolism and obesity (Ricoult and Manning, 2013). mTOR complex 1 (mTORC1) has been implicated in the regulation of adiposity since the discovery that genetically- or diet-induced obese mice display elevated activity of this complex in adipose tissue. Consequently, either WAT-specific knock-out of mTORC1 (Polak et al., 2008) or pharmacological mTORC1 inhibition (Houde et al., 2010) with rapamycin reduces adiposity and protect mice from diet-induced obesity. Additionally, mTORC1 is necessary for the maturation of 3T3-L1 preadipocytes and the activation of pro-lipogenic Sterol Regulated Element-Binding Protein (SREBP1).

Importantly, moderate, in contrast to full mTORC1 inhibition, aggravates HFD-induced obesity and adipogenesis raising the hypothesis that chronic mTORC1 overactivation in adipocytes is inhibitory to fat accretion and adiposity (Laplante et al., 2012). Accordingly, mice with constitutive mTORC1 activation in adipocytes induced by tuberous sclerosis complex (TSC1) deletion in differentiated, mature adipocytes significantly reduces visceral adiposity. Mechanistically, this phenomenon can be connected, at least in part, to a reduced adipocyte size and number, increased lipolysis, mitochondrial oxidative activity and browning (Magdalon et al., 2016).

Given the significance of c-Myc and mTOR in the regulation of growth, the presence of a direct regulatory link between them is probably crucial. Indeed, c-Myc is a direct repressor of TSC expression. In turn, TSC loss de-represses c-Myc protein, creating feed-forward regulatory loop (Schmidt et al., 2009). Downstream effectors of c-Myc-Cyclin D-CDK4/6- also phosphorylates and inactivates TSC2, resulting in mTORC1-activation. Conversely, inhibition of CDK4/6 led to decreased mTORC1 activity and reduced protein synthesis. Consistent with this, the anti-proliferative effect of CDK4/6-inhibition was reduced in cells lacking TSC2 (Romero-Pozuelo et al., 2020). It should be noted that the relevance of these various mechanisms in the context of human obesity and obesity-related comorbidities is unclear and requires further studies to elucidate the full-range of the dynamic molecular interaction. The possible clinical application of small-molecule Cyclin D-CDK4/6 inhibitors in metabolic disorders is another largely unexplored area (Fassl et al., 2022).

A mechanistic link between glucocorticoid signalling and c-Myc expression has been demonstrated. Dexamethasone, a synthetic glucocorticoid hormone, is a crucial adipogenic *in vitro* component that induces c-Myc transcription (Deisenroth et al., 2014). It appears that glucocorticoid stimulation is crucial for c-Myc induction in a concentration-dependent manner. Interestingly, dexamethasone treatment of 3T3-L1 preadipocytes was previously connected to the regulation of wingless-type MMTV integration site family (WNT) and transforming growth factor beta (TGF- β) genes and the induction of C/EBPα and PPARγ (Pantoja et al., 2008). This shows a molecular link between glucocorticoid signaling and c-Myc expression. Glucocorticoids might stimulate the differentiation of ASC into adipocytes, alter the lipid metabolism through reduced lipogenesis and increased lipolysis in mature adipocytes. These effects ultimately increase the adipose cell number, thereby leading to obesity, and inducing imbalance in the lipid metabolism of adipose tissue, which contributes to the development of IR (Ayala-Sumuano et al., 2013).

In fact, Cushing’s syndrome-related elevation of endogenous glucocorticoid cortisol is linked to obesity (Chaudhry and Singh, 2023). In addition, a characteristic side effect of long-term glucocorticoid therapy is an increase in central adiposity, which is partly attributed to an increase in hyperplasia inside adipose depots (Ayala-Sumuano et al., 2013).

These results are particularly intriguing because it has previously been demonstrated that glucocorticoids can cause lymphoid cell G1 arrest acting in part via inhibition of c-Myc expression. Similar effects have been reported in some fibroblastic cells (Ma et al., 2000). In fact, different cell types preferentially employ different modes of c-Myc control depending on their physiological status. Additionally, the cellular and tissue environment controls the functional activities of glucocorticoids. For instance, glucocorticoids are powerful anti-inflammatory agents in the immune system, whereas in the developing lung they are essential for normal maturation. If we understand the mechanisms of how this tissue specific activity is achieved, we should be able to develop more targeted therapeutic interventions with fewer side effects for a wide range of diseases that are either resistant to current therapy or for which glucocorticoid therapy produces unacceptable side effects (Feldman, 2009). Undoubtedly, the interaction between glucocorticoids and c-Myc is an area that need more study.

### Key points

• c-Myc is essential in ASCs adipogenesis.
• c-Myc inhibits the terminal stages of adipocyte differentiation.
• The SIRT1/c-Myc axis regulates both the quantity and functional integrity of adipocytes.
• Dexamethasone induces the transcriptional activity of c-Myc in adipocytes.

### MASLD- the nexus with obesity

MASLD, formerly known as non-alcoholic fatty liver disease (NAFLD), is linked to an increased risk of obesity (Rinella et al., 2023). The key feature of MASLD, steatosis, develops when the rate of hepatic FA intake from plasma and *de novo* synthesis is higher than the rate of FA oxidation and export (as triglycerides within very low-density lipoproteins (VLDL)) (Polyzos et al., 2019). Massive lipid accumulation in the liver leads to an imbalance of lipid metabolism inducing protein unfolding and ER stress, mitochondrial dysfunction and, ultimately, cell death that subsequently causes chronic inflammation and extended liver damage (Parthasarathy et al., 2020; Powell et al., 2021).

In Spain, it is estimated that MASLD affects, at least, 25.8% of the population aged between 15 and 85 years. The risk of developing more advanced stages of MASLD increases for patients older than 45 years. Moreover, the societal costs of this epidemics are estimated between €3.625 and €5.571 million (Higado, 2021).

Patients with MASLD frequently eat large quantities of processed foods heavy in fat, refined sugars, and carbohydrates, lead sedentary lifestyles, and engage in little physical activity. However, in addition to these exogenous or environmental factors, numerous other factors frequently influence the progression of MASLD and end-stage carcinogenesis. For instance, 42% of MASLD patients develop steatotic liver disease (SLD) and only 2.4%–12.4% finally develop liver cancer (White et al., 2012). Overall, large variety in the predisposition to develop MASLD demonstrates that among risk factors, endogenous (i.e., genetic) factors are particularly important (Guo et al., 2021).

Recent research from our lab has demonstrated that transgenic mice, bearing overexpression of c-Myc only in hepatocytes (Alb-myc^tg^) and fed a standard chow diet are predisposed to moderate obesity and aberrant hepatic lipid accumulation with ageing (Nevzorova et al., 2013; Guo et al., 2021).

Gene array analysis of the liver tissue of Alb-myc^tg^ mice consistently showed significant changes in FA metabolism. The overproduction of FA in c-Myc transgenic hepatocytes serves as a substrate and an inducer of P-450 (CYP)2E1 microsomal cytochrome FA oxidation systems (e.g., Cpt1, Adcam), which results in increased production of reactive oxygen species (ROS) and oxidative stress. The hepatic parenchyma becomes inflamed and infiltrated by immune cells triggered by ROS and lipid peroxidation products. Hepatic stellate cells (HSCs) are further activated by inflammatory cytokines released by immune cells (Arab et al., 2018). This prompts HSCs to produce collagen fibres and extracellular matrix (ECM) deposition in the hepatic parenchyma, which results in liver fibrosis (Nevzorova et al., 2013; Guo et al., 2021). Mechanistically, c-Myc overexpression in hepatocytes, caused by gene amplification or the inflammatory response to liver injury, initiates PDGF-B expression. The close proximity of dying PDGF-expressing hepatocytes pre-activates resident quiescent HSC, and encourages their transdifferentiation into myofibroblasts that produce collagen (Nevzorova et al., 2016; Zheng et al., 2017).

The excess FA produced by Alb-myc^tg^ liver is exported and transported to WAT for storage. This was linked to the enhanced deposition of VLDL particles high in triglycerides in the serum of middle-aged Alb-myc^tg^ animals (Alves-Bezerra and Cohen, 2017). As a result, compared to control littermates, c-Myc transgenic mice at 36 weeks of age gain significantly more weight, have higher BMIs, and have more WATs. Adiposity and low-grade WAT inflammation, which are demonstrated by the presence of macrophage crown-like structures (CLS), cause IR and hyperglycemia in transgenic mice (Bigornia et al., 2012; Zatterale et al., 2019). IR results in high level of blood glucose, and further contributes to metabolic disorders in the liver. Altogether, excessive c-Myc overexpression only in hepatocytes alters the body’s metabolism and causes moderate obesity, spontaneous hyperlipidemia, glucose intolerance, and mild steatohepatitis/fibrosis (Guo et al., 2021). Additionally, in various mouse MASLD (Fang et al., 2023) and hepatocellular carcinoma (HCC) models, c-Myc-induced metabolic alterations further increase hepatocarcinogenesis (Ma et al., 2000). In fact, this closely resembles human MASLD, where a combination of endogenous (such as oncogenes) and external (such as dietary habits) factors work together to promote the development of HCC. As proof of clinical significance, c-Myc expression is elevated in MASLD patients (Younes et al., 2022) and MASLD-related HCC (Freimuth et al., 2010; Guo et al., 2021).

In agreement with several studies (Akinyeke et al., 2013; Shen et al., 2018; Wang et al., 2021b), we reported (Guo et al., 2021) c-Myc inhibition by metformin. We demonstrated that Alb-myc^tg^ mice on a chow diet rich in metformin were resistant to obesity, showed modest improvements in hyperglycemia and dyslipidemia, and had less liver steatosis and fibrosis. We found that metformin had a strong inhibitory effect on *de novo* lipogenesis and particularly on SREBP1 expression in a Alb-myc^tg^ animals. Our observation is also consistent with prior report that c-Myc orchestrates the induction of lipogenesis, activates its master regulators SREBP1 and they collaborate to activate FA synthesis, and drive FA chain elongation from glutamine and glucose. Importantly, after inhibition of FA synthesis c-Myc-induced tumorigenesis is blocked and tumors regress in both xenograft and primary transgenic mouse models, revealing the vulnerability of Myc-induced tumors to the inhibition of lipogenesis (Gouw et al., 2019).

However, in our experimental conditions despite a notable improvement in steatohepatitis in Alb-myc^tg^ mice treated with metformin, we were unable to find any significant alterations in c-Myc-induced hepatic proliferation (Guo et al., 2021). However, several studies indicate that metformin can lower the risk of cancer (including HCC) in people with T2DM in a dose-dependent manner (Hassan et al., 2010; Bo et al., 2012; Chen et al., 2013).

There is also evidence that statins might lower the frequency of HCC. In fact, statins have anti-inflammatory and immunomodulatory properties; they prevent the generation of cell growth mediators and encourage programmed cell death (Islam et al., 2020). It has been showed (Rao and Rao, 2021) that simvastatin, atorvastatin, and lovastatin prevent c-Myc activation, which in turn inhibits growth of cancer cells (Shachaf et al., 2004). MiR-33b, a specific inhibitor of c-Myc, is often missing in medulloblastomas. Its overexpression causes c-Myc downregulation. It has been demonstrated that lovastatin elevated mi-R-33b expression, which in turn inhibited cell proliferation (Takwi et al., 2012).

Tumour growth in orthotopically xenografted cells is also inhibited by lovastatin administration. The objective of statins as a pharmacological modulator of c-Myc via miRNA-based treatments may benefit from this research. This indicates that statins can be used as a pharmacological modulator of c-Myc via miRNAbased therapeutics (Di Bello et al., 2020).

Despite constant exposure to microbial-derived and food products from the gut, the liver is a crucial immune organ that is sterile and tolerogenic. One of the largest populations of T cells in liver are mucosal-associated invariant T (MAIT) cells, an innate T-cell that may quickly respond to stimulation, start proliferation, and produce cytokines and lytic molecules (Kurioka et al., 2016). MAIT cells are essential for the host’s defence against bacterial and viral infections. c-Myc is required for the proliferation of MAIT cells. Upon activation, MAIT cells significantly upregulate c-Myc target proteins, regulating amino acid transport, glycolysis, and cell division. Obesity has been linked to impaired MAIT cell proliferation and reduced functional responses due to an impaired Myc-SLC7A5-glycolysis metabolic axis. Reduced MAIT cell proliferation in obese persons may increase host sensitivity to infection and malignancies (Kedia-Mehta et al., 2022).

### Key points

• Middle-aged transgenic mice with c-Myc overexpression in hepatocytes (Alb-myc^tg^) develop mild obesity and abnormal hepatic lipid accumulation upon standard chow feeding.
• Metformin partly attenuates the spontaneous obesity and MASLD in Alb-myc^tg^ mice.
• c-Myc overexpression is a hallmark of MASLD and MASLD-related HCC, highlighting the pivotal role it plays in the development of the disease.
• c-Myc is required for MAIT cells proliferation and is dysfunctional in obesity.

### *β*-CELLS of the langerhans islets–the pancreatic player in obesity-linked T2DM

Obesity-linked T2DM is a disease of encompassing IR in combination with pancreatic *β*-cell dysfunction (Abdullah et al., 2010). The risk of T2DM is 93 times greater in patients with a BMI over 35 kg/m^2^ (Barnes, 2011). Obesity is nowadays an epidemic of unforeseen proportions. In 2000, 9% of people in Spain had T2DM, while 15% of the population was obese. If the trend continues, 12% of the nation’s population will have T2DM by 2030 (Huerta et al., 2013).

In early stages of obesity, *β*-cells increase their mass and function to compensate for peripheral IR. However, if the condition becomes more chronic and severe, the adaptability of *β*-cell declines, resulting in a reduction in *β*-cell mass. TD2M arises if the endocrine pancreas fails to secrete sufficient insulin to handle the metabolic demands caused by *β*-cell secretory disfunction and/or relative decreased *β*-cell mass (Chen et al., 2017). The fact that obesity-linked T2DM develops in only 25%–30% of obese individuals raises the possibility that a genetic predisposition plays a role in individual susceptibility (Lingohr et al., 2002).

A dynamic balance between cellular growth and death determines the number of *β*-cells required to maintain proper glucose homeostasis in mammals (Rhodes, 2005; Rosselot et al., 2021). Pancreatic *β*-cell mass is increased due to at least three mechanisms: i) *β*-cell neogenesis (differentiation from precursor cells); ii) *β*-cell proliferation; and iii) *β*-cell hypertrophy (increased cell size). In turn, *β*-cell death, primarily by apoptosis or *β* -cell atrophy (decreased cell size), reduces the number of *β*-cell (Bonner-Weir, 2000; Ackermann and Gannon, 2007; Saisho et al., 2013).

The signal transduction pathways controlling the proliferation and survival of *β*-cells hold particular significance (Lingohr et al., 2002). c-Myc seems to have an important physiological impact on these processes (Jonas et al., 2001). In *β*-cells, c-Myc is typically expressed at very low basic levels. However, in response to glucose, it may transiently and moderately rise, promoting the replication of *β*-cells (G1/S transition). The proliferative silence of *β*-cells can be successfully overcome by the ectopic expression of c-Myc. Even in the absence of replication, c-Myc plays a significant role in cell growth (size) (Collier et al., 2003). Therefore, c-Myc transiently and moderately increases during the growth of *β*-cells, acting as a metabolic regulator (Karslioglu et al., 2011).

Interestingly, plasma insulin does not induce c-Myc in pancreatic islets. Exogenous insulin added to primary rat *β*-cells failed to alter c-Myc expression, as demonstrated by numerous *in vitro* and *in vivo* experiments (Elouil et al., 2005). Additionally, the inhibitor clonidine reduces insulin release but does not stop the rise in c-Myc mRNA caused by glucose (Plant et al., 1991). Therefore, during hyperglycemia, glucose rather than insulin induces elevated c-Myc levels.

Chronic hyperglycemia, or high blood glucose levels, is the definition of T2DM. Consequently, *β*-cell exposed to high glucose concentrations in diabetic conditions. Moreover, pancreatic *β*-cells have substantially greater glucose concentrations than many other cell types because they are surrounded by a dense network of fenestrated capillaries that facilitates better blood glucose exchange (Veld and Marichal, 2010). Thus, c-Myc expression in *β*-cells *in vivo* is significantly impacted by hyperglycemia (Rosselot et al., 2021).

Short-term HFD feeding in young mice increases body weight, IR and glucose intolerance. After HFD feeding, c-Myc protein abundance in *β*-cell is increased and compensatory *β*-cell proliferation, expansion and cell function are induced. Mechanistically, c-Myc upregulation in pancreatic islets is mediated by a PKCζ, ERK1/2, mTOR, and PP2A pathway and target genes mediate cell cycle pathways (Rosselot et al., 2019). Consistently, glucose intolerance and hypoinsulinemia after short HFD feeding in mice with c-Myc deficiency in *β*-cells indicates that c-Myc is crucial for the adaptive response of islets to acute metabolic insults (Rosselot et al., 2021).

Due to restrictions in cell replication, adults’ ability to increase their *β*-cell mass is limited. In contrast, the proliferation of neonatal functionally immature *β*-cells is robust. Juvenile *β*-cells undergo functional maturation in the early postnatal period and develop the glucose-responsive, insulin secretory phenotype. Importantly, c-Myc regulates *β*-cell proliferation and immaturity. Rodent juvenile islets have elevated levels of c-Myc, which promotes the rapid proliferation of neonatal *β*-cells. The number of proliferating cells in postnatal stages decreases when endogenous c-Myc in *β*-cells is deleted *in vivo* (Rosselot et al., 2021)*.* Consistently, stabilisation of c-Myc not only encourages replication but also directs *β*-cells towards functionally immature phenotypes, simulating postnatal *β*-cell functionality. Ablation of c-Myc in neonatal *β*-cells consistently results in impaired cell cycle progression and proliferation, and reduced functional *β*-cell mass (Puri et al., 2018). *In vitro* studies using rodent and human cell lines, have revealed that the bidirectional shift between fully functional, mature, non-proliferative *β*-cells and proliferative, functionally immature *β*-cells is reversible (Scharfmann et al., 2014). Overall, the ability of *β*-cells to replicate impairs its function. However, if just a small percentage of cells replicate, as happens in adult islets, transitory loss of function in *β*-cells is adequate. When a larger fraction of *β*-cell divides, overall *β*-cell function deteriorates and the insulin processing and release are dysregulated (Liu and Hebrok, 2017; Puri et al., 2018). Consistently, the analysis of the active chromatin marks on human genomes confirms that c-Myc activity is increased at younger ages (Puri et al., 2018).

In both humans and rodents, the ability of *β*-cells to replicate decreases with age (Tschen et al., 2009). Ageing reduces both the adaptive responses to mitogens like HFD as well as the basic proliferative mitogenic response of *β*-cell. In contrast to young mice, older animals fed with HFD had diminished c-Myc action in their islets. Mechanistically, epigenetic-mediated c-Myc resistance restricts, at least partially, the adaptive proliferation of *β*-cell in the context of increased insulin demand during aging (Rosselot et al., 2021). “c-Myc resistance” in metabolically stressed aged *β*-cells can possibly explain why aging population are generally more prone to developing T2DM (Rosselot et al., 2021).

Overall, c-Myc is essential for the regeneration of for *β*-cells under basal or metabolically stressed conditions. From a therapeutic perspective, agents that promote human *β*-cell replication may be helpful if such activity is reversible. This, of course, provoke the interest for c-Myc as potential therapeutic target in regenerative therapy for diabetic patients.

Mice with constitutive or inducible transgenic overexpression of c-Myc in *β*-cells were created by different groups (Pelengaris and Khan, 2001; Laybutt et al., 2002; Pelengaris et al., 2002; Cano et al., 2008; Murphy et al., 2008) to clarify whether c-Myc might be able to stimulate proliferation with therapeutic potential. Although remarkable *β*-cell proliferation was induced by c-Myc overexpression, this proliferation was very apparent, brief, and obviously carcinogenic, making these results disappointing. Moreover, *β*-cell proliferation was associated by immediate *β*-cell dedifferentiation and/or death, leading to diabetes. Indeed, in pIns-c-MycER^TAM^ mice upon tamoxifen stimulation *β*-cell destruction was so extensive that these transgenic mice were even used as a model of complete *β*-cell ablation (Cano et al., 2008). In islets from c-Myc-overexpressing mice, gene expression analysis revealed stabilisation of p53 and activation of the intrinsic apoptotic and DNA-damage checkpoint mechanisms (Cheung et al., 2010; Robson et al., 2011).

Overall, studies with transgenic mice show that high (estimated in the 20- to 50-fold range) and persistent overexpression of c-Myc in *β*-cells results in cell dysfunction and death. Certainly, c-Myc plays a critical role in glucotoxicity-induced *β*-cell death in chronic hyperglycemia and diabetes (Karslioglu et al., 2011).

Despite the fact that excessive c-Myc expression is harmful for *β*-cells, low physiological levels of Myc are necessary for normal *β*-cell functionality. Recent research has demonstrated that the usage of harmine (β-carboline alkaloid) mildly upregulates c-Myc expression and stimulates adult human *β* -cell cycle entry at rates that are in the physiological and potentially therapeutic range (Wang et al., 2015). In addition, harmine combined with GLP-1R agonists (Ackeifi et al., 2020) or TGFß inhibitors dramatically increases human *β*-cell proliferation (5%–8%), indicating that combination treatments targeting multiple signalling pathways may be more effective for islet regeneration in T2DM patients (Wang et al., 2019). Further approaches to optimize the use of harmine (Title et al., 2022). and the development of methods to specifically target *β*-cells, present an important translational challenge (Rosselot et al., 2021).

The great majority of research on *β*-cell proliferation was conducted on rodents, which has increased our understanding of murine rather than human *β*-cell replication. However, there are significant differences between human and rodent islets in terms of their function, composition, structure, and in proliferative capacity. These differences highlight the need to focus future research on human islets proliferation and partially explain why most substances that have been shown to increase *β*-cell proliferation in rodent islets have not been successful in humans (Wang et al., 2021a).

### Key points

• Glucose rapidly stimulates c-Myc expression in *β*-cells.
• c-Myc is an inverse dual regulator of *β*-cell maturation and proliferation.
• Proliferation of *β*-cell is induced by mild physiologic upregulation of c-Myc.
• High and persistent c-Myc overexpression results in *β*-cells dysfunction and cell death.

### Intestine–the gatekeeper of diet-induced obesity

While unhealthy diets and sedentary lifestyles synergistically with polygenetic risks represent major causes of obesity, a big plethora of data suggest that the intestine also plays a part as a crucial organ participating in glucose and lipid metabolism (Hur and Lee, 2015). In fact, the gastrointestinal tract is the first organ to be exposed to dietary components. Unhealthy diets interact with gut microbiota (GM) to promote early intestinal inflammation which favor obesity and IR. The altered epithelial permeability, bacterial products translocation, upregulation of proinflammatory cytokines and intestinal endocrine hormones are the main pathophysiological mechanisms (Ding and Lund, 2011).

Epithelium in the gastrointestinal tract has a precise architecture, formed by invaginations, or crypts, and finger-like lumenal protrusions, or villi. These ‘‘folds’’ create an enormous surface area, allowing efficient nutrient absorption from the intestinal space. The self-renewing intestinal stem cells (ISCs) are located in crypts and intervilli areas and continuously produce a population of rapidly proliferating progenitor cells that migrate towards the intestinal lumen. As they migrate, cells undergo cell cycle arrest and commit to different cell lineages by terminal differentiation (Marshman et al., 2002). In the small intestine and colon, cells develop into three functional cell types: 1. the predominant enterocyte; 2. the mucus-secreting Goblet cells and; 3. the peptide hormone secreting enteroendocrine cells. Moreover, cells that descend to the base of the crypt in the small intestine convert into the Paneth cells, the fourth cell type. Differentiated cells carry out their specific tasks and then after induction of apoptosis, discarded into the lumen (Allaire et al., 2019).

c-Myc plays an important role in regulating homeostasis, proliferation, differentiation, and transformation in the adult gut (Marshman et al., 2002; Sancho et al., 2003). All intestinal epithelial cells (IEC) of the crypt-villus unit, with the exception of Paneth cells, express c-Myc. Cell cycle arrest and the upregulation of the cell cycle inhibitor inhibitor p21^cip/waf^ coincide with the differentiation of proliferative IEC, which is also accompanied by a decrease in c-Myc expression (Pinto et al., 2003). In gastric and colonic tissue c-Myc overexpression is associated with inflammation as well as with potentially neoplastic hyperproliferative states. Overall, c-Myc is crucial for maintaining control of intestinal crypt homeostasis and cellular proliferation. Wnt signalling pathway is a most likely upstream regulator that controls these processes (Bettess et al., 2005). Inhibition of the Wnt pathway in the intestinal mucosa of mice, via overexpression of Dkk1 inhibitor leads to diminished number of crypts, concomitant with a loss of cell proliferation (Kuhnert et al., 2004). In turn, the loss of c-Myc expression and a rise in p21^cip/waf^ expression are linked to a reduction in proliferation (Pinto et al., 2003).

In adult mice, c-Myc is dispensable for homeostasis and IEC proliferation but essential for the development of intestinal crypts. Tamoxifen-inducible depletion of c-Myc in the mucosa of adult and juvenile mice at the onset of crypt morphogenesis causes the failure to form normal numbers of crypts in the small intestine. Yet, mice are able to recover from this insult and form and maintain a normal IEC and without compensation by n-Myc or l-Myc (Bettess et al., 2005). Knock-out mice of c-Myc specifically in IEC under the control of a cre promoter (c-Myc^ΔIE^) die before adulthood. However, c-Myc^ΔIE/+^ heterozygous mice, with reduced c-Myc expression, are complete viable, metabolically fit and display normal intestinal morphology (Luo et al., 2021).

HFD overnutrition, induces IEC proliferation by stabilizing *β*-catenin. Activation of the *β*-catenin pathway stimulates the expression of downstream genes including cyclin D, that, in turn, prompts IEC proliferation, further contributing to the increased absorption of nutrient and obesity development (Petit et al., 2007; Mao et al., 2013). c-Myc is a *β*-catenin target gene and key TF regulating the cell cycle. Hence, a significant induction of intestinal c-Myc expression was shown in C57BL/6N mice fed with HFD (Luo et al., 2021). Higher c-Myc expression was also seen in the distal ileum biopsies of the obese patients, which is consistent with mouse results. Additionally, c-Myc expression had a positive correlation with BMI and ALT levels in serum (Luo et al., 2021).

Importantly, c-Myc^ΔIE/+^ heterozygous mice are protected against HFD-induced obesity, IR, hepatic steatosis and fibrosis. Mechanistically, reduced expression of c-Myc in the intestine increases ChREBP and GLUT2/SGLT1 expression, thus promoting glucagon-like peptide-1 (GLP-1) production and secretion. GLP-1 is one of the crucial gut-derived peptide hormones that stimulates insulin secretion and thereby controls glucose homeostasis (Andersen et al., 2018). Increased GLP-1 synthesis in c-Myc^ΔIE/+^ mice improves IR and boosts insulin release in response to glucose (Luo et al., 2021).

Furthermore, intestinal c-Myc enhances levels of ceramides by targeting Cers4, a crucial enzyme of *de novo* ceramides synthesis (Luo et al., 2021). Ceramides are bioactive lipids that have an impact on inflammation, apoptosis, oxidative and ER stress, IR, and energy metabolism. There are three different ways to synthesize ceramides: the *de novo* pathway, the sphingomyelinase pathway and the salvage pathway (Aburasayn et al., 2016). Through genetic or pharmacological modification of ceramide biosynthesis and catabolism in mouse models, a crucial role for ceramides in metabolic disorders was demonstrated (Chaurasia et al., 2016). Mice with decreased intestinal c-Myc expression are resistant to dietary-induced metabolic disorders, and this resistance has a strong correlation with lower blood ceramide levels (Luo et al., 2021).

Whether the c-Myc–GLP-1 pathway and the c-Myc–ceramide pathway in the intestine co-operate with each other is unknown and requires further investigation. Besides, the roles of intestinal cell-type-specific c-Myc in metabolic diseases are worth investigating thoroughly in the future.

Interestingly, oral administration of 10058-F4, a c-Myc-Max interaction inhibitor, to obese mice greatly reduces obesity, IR, steatosis, and liver fibrosis. The metabolic benefits are mostly mediated by changes in GLP-1 and ceramide levels. Taking into account the absence of the current therapy for MASLD, the intestinal c-Myc pathway may be an attractive new area of investigation. Given the lack of a current MASLD treatment, research into the intestinal c-Myc pathway would be an appealing new field (Luo et al., 2021).

The dynamic equilibrium between ISC self-renewal and differentiation is crucial for maintaining intestinal homeostasis. Infiltration of macrophages and other immune cells as well as a persistent low-grade inflammation are linked to obesity. Macrophages infiltrating in the colonic mucosa contribute directly to the production of colonic TNF-α. Additionally, TNF-α secreted by the immune cells in the adipose tissue is also found circulating in the colonic mucosa. TNF-α can induce the phosphorylation of GSK-3 and reduce the Apc complex’s ability to phosphorylate and degrade *β*-catenin. In turn, this triggers the production of the Wnt target genes c-Myc and cyclin D1, which in turn promotes the growth of ISCs and the development of obesity-related colorectal cancer (Liu et al., 2012). Although the particular mechanisms causing the low-grade inflammation caused by obesity are not entirely understood, increased palm oil consumption may be one of the initial causes of gastrointestinal alterations (Ghezzal et al., 2020).

In addition to being a complex of various organs and systems, the human body also carries more than 500–1000 different species of microbes. Numerous studies have been lately done on the complexity and variety of the GM in relation to human health and disorders. Growing evidences have underlined the importance of GM dysbiosis for the development and progression of metabolic diseases and obesity-related carcinogenesis (Kobyliak et al., 2016).

A thinner mucous layer, uneven localization of tight junction proteins (TJP), an abnormal immunological response involving immunoglobulin A (IgA), and antimicrobial peptides like lipopolysaccharides (LPS) can all contribute to intestinal disbiosis in obese people. Collectively, these defects cause LPS leakage, which eventually leads to TLR4/MyD88 and NF-κB activation and inflammation (Singh et al., 2023).

Numerous tumorigenic pathways, including members of the STAT family (particularly STAT3), can be stimulated by inflammation. STAT3 enhances the expression of anti-apoptotic genes, which lead to cellular survival and growth by promoting cyclin D family members and c-Myc. Therefore, GM obesity-related alterations may accelerate the development of colorectal cancer (CRC) by triggering inflammatory pathways (Kolb et al., 2016; Singh et al., 2023).

The identification of specific microbial taxa associated with obesity and T2DM still remains difficult. However, specific bacteria may be essential in triggering metabolic inflammation during the course of a disease. For example, HFD results in the enrichment of the Enterobacteriaceae *family*, which is predominately represented by *Escherichia coli* (*E. coli*), and has a strong association with poor glucose homeostasis (Ju et al., 2023). Certain *E. coli* strains with the polyketone acid synthetase (pks) island have the ability to produce the colibactin toxin and cause a proliferative effect linked to colorectal cancer (CRC). c-Myc is activated in pks + *E. coli*-infected CRC cells, which causes miR-20a-5p upregulation. Upregulation of miR-20a-5p can subsequently cause the translational silencing of target SENP1. SENP1 is a crucial enzyme that prevents the modification of p53 patterns, which is a key regulator of cellular senescence. The senescence of IEC in pks + *E. coli*–infected CRC cells stimulates the secretion of growth factors, essential for the initiation of tumour growth (Xing et al., 2022).

The secretion of different metabolites plays a major role in mediating the beneficial effects of GM. Acetate, propionate, and butyrate are three small organic metabolites called short-chain fatty acids (SCFAs) that are formed when resistant starch and dietary fibres are fermented. SCFA showed a variety of beneficial effects on immunological responses, energy metabolism, and intestinal homeostasis. Obesity and metabolic disorders have been associated with an abnormal SCFAs production. Butyrate is one of the SCFAs that has lately gained attention due to its ability to alleviate obesity and its associated comorbidities. Lower butyrate-producing microbial abundance in humans has been linked to a higher risk of metabolic disorders, demonstrating its potency in obesity prevention (Coppola et al., 2021). Interestingly, butyrate rapidly suppresses c-Myc levels in human CRC cells, which, in turn, reduces the levels of the miR-17–92 cluster miRNAs and decreases angiogenesis, metastasis, and cell proliferation (Hu et al., 2015). These indicate that butyrate may decrease the progression of CRC by altering the expression of tumour miRNAs, which causes changes in a number of critical signalling pathways, including c-Myc (Yuan and Subramanian, 2019).

### Key points

• c-Myc is crucial for the control of homeostasis and the proliferation of IEC.
• Improvements in HFD-induced obesity, IR, and steatohepatitis are seen in mice with intestine-specific reduction of c-Myc.
• Obesity-associated changes of GM may activate c-Myc and cause progression of the colorectal cancer.

## Conclusion and future perspectives

Nowadays, due to its alarming prevalence, obesity has emerged as the most dangerous nutritional disease and a significant health risk for people. In order to regulate the occurrence of this disease, it is necessary to control the nutritional habits and avoid sedentary life style. Yet the development of obesity is inseparable from epigenetics, which together with genetic factors play a pivotal role in its pathogenesis. Various TFs are critical participants in obesity and associated metabolic disorders such as T2DM and MASLD (Huang et al., 2018). In the present review, we show, that c-Myc is an important player in the multisystemic pathogenesis of obesity and its dysregulation is involved in inflammatory, metabolic, proliferative disorders in multiple organs. c-Myc is a typical moonlighting protein - a protein with a great number of functions that is unrelated and independent to each other. In WAT, liver, intestine, and pancreas, it controls the expression of genes involved in cell proliferation and growth, apoptosis, organogenesis, and metabolism. Additionally, it influences the nucleus’ general structure, gene and microRNA expression, and genomic amplification (Lv and Lei, 2021).

Consequently, targeting c-Myc may open up novel strategies to combat obesity. However, the inactivation of a master regulator protein essential to normal cell proliferation and survival is thought to have substantial adverse effects, making c-Myc a dangerous therapeutic target (Dang et al., 2017). For instance, c-Myc is essential for potential regeneration strategies of the *β*-cells under baseline or metabolically stressed conditions. Furthermore, it is crucial for controlling intestinal cellular proliferation. All of this points to the urgent need for targeting c-Myc activity that is more cell-type specific, and taking into account the negative effects of its aberrant expression.

Over the last decades, several approaches have attempted to suppress c-Myc directly or indirectly at all levels of its regulation. Omomyc, for instance, has demonstrated promising properties in pre-clinical testing; it can induce apoptosis (Soucek et al., 2002) in cancer cells but not in normal cells, prevent proliferation and invasion (Beaulieu et al., 2019), stop the communication between the tumour and its microenvironment and recruit immune cells to the tumour site (Demma et al., 2019; Madden et al., 2021). Omomyc is a 90 amino acid Myc mini-mutant that comprises the bHLH-LZ domain and competes with c-Myc, n-Myc and l-Myc for binding to DNA and preventing the transcription of the target genes (Soucek et al., 1998). *In vivo* Omomyc-mediated c-Myc inhibition resulted in sustained tumour regression and a strong anti-proliferative effect, with no negative effects on healthy tissue. Despite its short effective half-life, a phase I/II clinical trial started in 2021 making Omomyc (OMO-103) the first direct c-Myc inhibitor to reach clinical phase studies in patients with advanced solid tumors including non-small cell lung, colorectal and triple-negative breast cancer (Demma et al., 2019). Altogether, Omomyc taught us that c-Myc inhibition is a practicable approach and a safe and effective therapeutic strategy (Masso-Valles and Soucek, 2020). In a future, Omomyc and related polypeptide inhibitors of c-Myc function can potentially be a viable alternative therapeutic strategy for a wide variety of c-Myc-related disorders in obesity (Madden et al., 2021).

**FIGURE 1:**
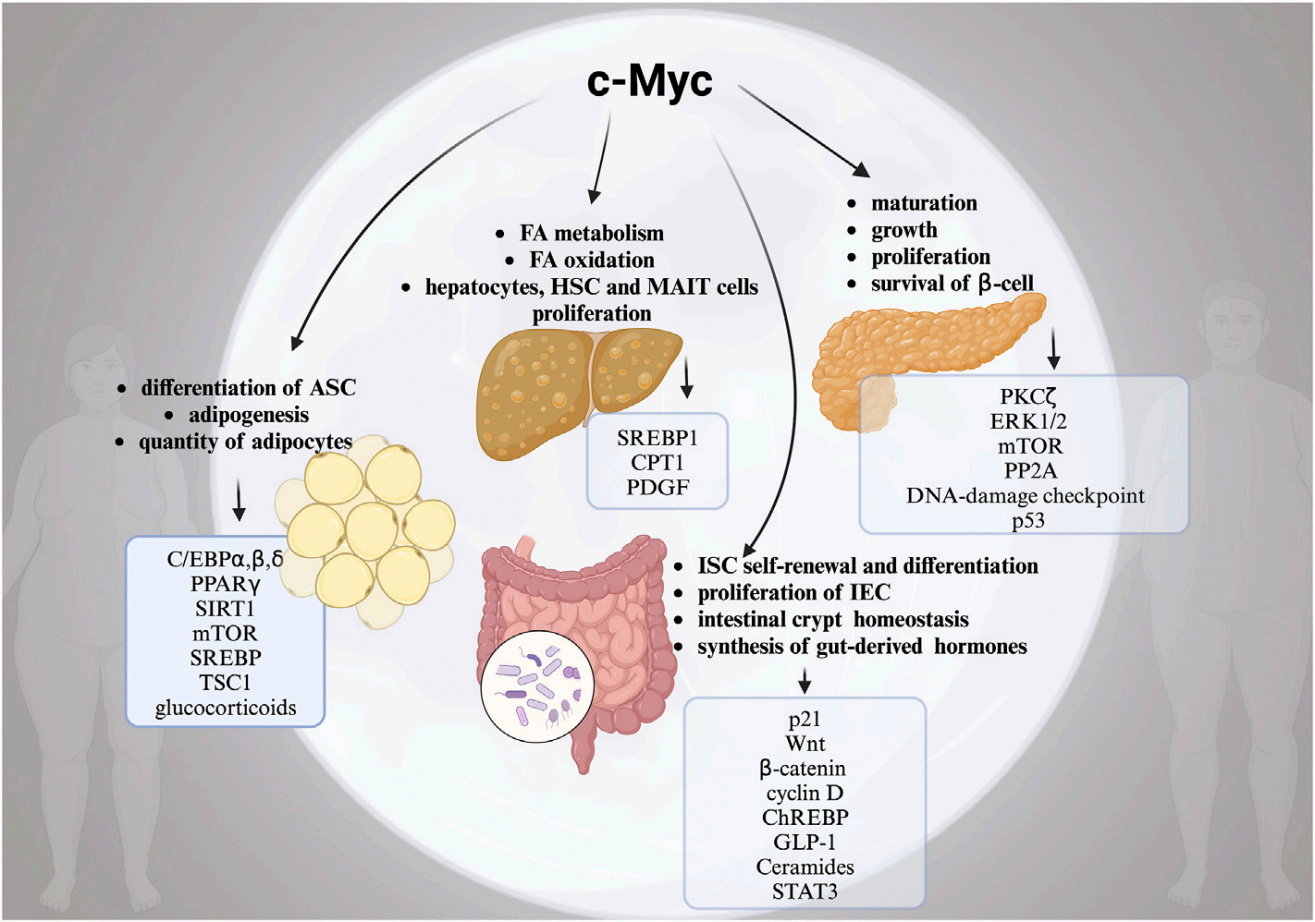
The complex role of moonlighting c-Myc for the development of obesity. Alterations in multiple c-Myc-related pathways in white adipose tissue (WAT), pancreas, liver and intestine in obesity. *Created with BioRender.*

Another significant aspect is that obesity is a risk factor for a number of serious malignancies, such as CRC, HCC, and pancreatic cancer. In addition to altered FA metabolism, ECM remodelling, IR, GM dysbiosis, changed microenvironment, poor progenitor maturation, and chronic inflammation, the link between obesity and the development of cancer is not fully understood (Pati et al., 2023). As we summarized in this review, c-Myc actually plays a crucial part in each of these processes, contributing to multisystemic pathogenesis of obesity (Figure 1). Although the specific mechanisms for c-Myc and high risk of obesity and cancer are elusive, the correlation is definite. Hence, the evaluation of the molecular mechanisms underlying the dangerous liaisons between c-Myc, obesity, and obesity-associated cancers are of high priority for the identification of novel therapeutic targets. Importantly, c-Myc can be used as a diagnostic target to identify the “high risk” obese patients who require serious consideration for preventative measures like routine screening and personalized counselling.

## Figures and Tables

**Table:** 

**ASCs**	Adipose stem cells
**ATP**	Adenosine triphosphate
**Alb-myc** ^**tg**^	transgenic mice with overexpression of c-Myc in hepatocytes under the control of albumin promoter
**BMI**	Body mass index
**c-Myc** ^**ΔIE**^	Knockout mice of c-Myc specifically in intestinal epithelial cells under the control of a cre promoter
**C/EBP**	CCAAT/enhancer binding protein
**CRC**	Colorectal cancer
**CLS**	Crown-like structures
**CDK**	Cyclin-dependent kinase
**NAFLD**	Non-alcoholic fatty liver disease
**ECM**	Extracellular matrix
**ER stress**	Endoplasmic reticulum stress
**FA**	Fatty acids
**GLP-1**	Glucagon-like peptide-1
**GM**	Gut microbiota
**HSCs**	Hepatic stellate cells
**HCC**	Hepatocellular carcinoma
**HFD**	High fat diet
**IR**	Insulin resistance
**IEC**	Intestinal epithelial cells
**ISCs**	Intestinal stem cells
**LPS**	Lipopolysaccharides
**mTORC**	Mammalian target of rapamycin complex
**MAIT**	Mucosal-associated invariant T
**MASLD**	metabolic dysfunction-associated steatotic liver disease
**NF-κB**	Nuclear factor kappa b
**PPARγ2**	Peroxisome proliferator-activated receptor γ2
**PGC-1**	Peroxisome proliferator-activated receptor gamma coactivator 1
**pIns-c-MycER** ^**TAM**^	Tamoxifen inducible transgenic mice with overexpression of c-myc in pancreatic *β* cells under the control of an insulin promoter
**Pks**	Polyketone acid synthetase
**ROS**	Reactive oxygen species
**SCFAs**	Short-chain fatty acids
**SREBP1**	Sterol Regulated Element-Binding Protein
**SIRT1**	Sirtuin-1
**SLD**	Steatotic liver disease
**TGF-β**	Transforming growth factor beta
**TJP**	Tight junction proteins
**TF**	Transcription factor
**TG**	Triglycerides
**TSC1**	Tuberous sclerosis complex
**T2DM**	Type 2 diabetes mellitus
**VLDL**	Very-low-density lipoprotein
**WNT**	wingless-type MMTV integration site family
**WAT**	White adipose tissue
**WHO**	World Health Organization
